# Calcium Ion Sensors with Unrivaled Stability and Selectivity Using a Bilayer Approach with Ionically Imprinted Nanocomposites

**DOI:** 10.3390/nano15100741

**Published:** 2025-05-15

**Authors:** Antonio Ruiz-Gonzalez, Roohi Chhabra, Xun Cao, Yizhong Huang, Andrew Davenport, Kwang-Leong Choy

**Affiliations:** 1 Institute for Materials Discovery, University College London, London WC1E 7JE, UK; a.gonzalez.16@ucl.ac.uk; 2 UCL Department of Renal Medicine, Royal Free Hospital, University College London, London WC1E 6BT, UK; roohi.chhabra@nhs.net (R.C.); yzhuang@ntu.edu.sg (Y.H.); andrewdavenport@nhs.net (A.D.); 3 School of Materials Science and Engineering, Nanyang Technological University, 50 Nanyang Ave, Singapore 639798, Singapore; caox0015@e.ntu.edu.sg; 4 Division of Natural and Applied Sciences, Duke Kunshan University, Suzhou 215316, China

**Keywords:** ion-selective electrode, biosensor, electrochemical sensor, calcium sensor, ion imprinting, molecular imprinting

## Abstract

Calcium ion sensors are essential in clinical diagnosis, particularly in the management of chronic kidney disease. Multiple approaches have been developed to measure calcium ions, including flame photometry and ion chromatography. However, these devices are bulky and require specialized staff for operation and evaluation. The integration of all-solid-state ion-selective determination allows the design of miniaturized and low-cost sensing that can be used for the continuous monitoring of electrolytes. However, clinical use has been limited due to the low electrochemical stability and selectivity and high noise rate. This manuscript reports for the first time a novel miniaturized Ca^2+^ ion-selective sensor, developed by using a two-layer nanocomposite thin film (5 µm thick). The device consists of functionalized silica nanoparticles embedded in a poly(vinyl chloride) (PVC) film, which was deposited onto a nanoporous zirconium silicate nanoparticle layer that served as the sensing surface. Systematic evaluation revealed that perfluoroalkane-functionalized silica nanoparticles enhanced Ca^2+^ selectivity by minimizing K^+^ diffusion, confirmed by both potentiometric measurements and quartz microbalance studies. The final sensor demonstrated a super-Nernstian sensitivity of 37 mV/Log[Ca^2+^], a low signal drift of 28 µV/s, a limit of detection of 1 µM, and exceptional selectivity against Na^+^, K^+^, and Mg^2+^ ions. Long-term testing showed stable performance over three months of continuous operation. Clinical testing was conducted on patients with chronic kidney disease. An accurate real-time monitoring of electrolyte dynamics in dialysate samples was observed, where final concentrations matched those observed in physiological conditions.

## 1. Introduction

Calcium-selective electrodes are ubiquitously employed in all scientific fields. They are crucial in diagnosis and management due to the involvement of calcium ions in multiple physiological processes and both acute and chronic health conditions, including cardiovascular and kidney diseases [1], and they are one of the main parameters measured in the assessment of water hardness [2], and essential in the determination of water quality [3]. In addition, they have been proven to be a useful tool in structural monitoring due to their high presence in concrete mixtures [4], and agriculture assessing plant and crop health [5]. The conventional approach in this field is the use of a plasticized PVC membrane with one or more ionophores, molecules that can bind the target analyte in a selective and reversible manner [6]. 

The composition of the sensing film, especially in terms of the choice of polymer and plasticizer, plays a pivotal role in the selectivity of the sensors. In particular, the dielectric properties of the plasticizer have been proven to have an impact on the performance of the electrodes by reducing the energy associated with the ion transfer process from the sample to the membrane phase [7]. In the case of divalent ions for example calcium, high dielectric plasticizers such as o-nitrophenyl octyl ether are often required to ensure reliable performance of the sensors [8,9]. However, these compounds are not biocompatible, limiting their applications in wearable technologies. 

The selectivity of ion sensors for calcium detection has as yet not been fully achieved, and the devices are typically subjected to interference by other ions [10]. To increase the selectivity of these calcium ion sensors, new ionophores and molecular receptors have been proposed. Zahran et al. [11] developed a cyclosporine-based PVC sensitive film with high selectivity towards diluted calcium ions. Although the sensors showed a high preference towards the detection of calcium ions, the selectivity of the devices was relatively low, with high interference from cesium and rubidium ions in solutions. Moreover, ionophore-based ion-selective electrodes are typically subjected to performance loss over time due to leaching of the active components over time [12]. Thus, there is an unmet need for the development of new selective sensing materials for the detection of calcium ions in solutions.

The interest in the use of microporous materials such as zeolites as electrochemical probes for the selective adsorption and detection of ions has risen within recent years. These materials combine the advantages of a high selectivity and the possibility of tailoring the pore properties to create homogeneous surfaces for the ionic exchange, while showing a higher stability, and an improved biocompatibility [13]. Arvand et al. [14] demonstrated the superior performance of zeolite-modified electrodes in the detection of diluted ions. This material exhibited high stability and selectivity towards Cs+ and could be employed for its detection in water samples. In addition, zirconium cyclosilicates have been employed as a highly stable and biocompatible material for the adsorption of potassium ions in solutions, showing at least 25 times more selectivity towards potassium than sodium ions in solution [15].

Recent advances in nanotechnology can potentially expand the capabilities of current ion sensors improving their selectivity and the signal stability of the devices. In particular, the integration of nanomaterials within the polymeric membranes can improve the diffusion of selective ions towards plasticized polymeric films and offer ionic sites for the interaction of the electrolytes. Tong et al. [16] demonstrated this concept by improving the ionic conduction of polymeric films of sulfonated poly (2,6-dimethyl-1,4-phenyleneoxide) using silica nanoparticles containing sulfate functionalization. In addition, functionalized nanoparticles containing sulfonated groups have been reported in ionic exchange matrices for the separation of Pb^2+^ and Cd^2+^, enhancing the absorption rates of the polymers [17]. Recently, the use of sensing films incorporating PVC-functionalized silica nanoparticles have led to the most electrochemically stable device reported to date, with a noise rate down to 1.3 µ h^−1^ [18], and was reported also to function under high flow rate conditions. However, there is a lack of systematic studies focused on the effect of the interface between functionalized silica nanoparticles and the polymer films. This study would allow the development of highly selective and stable sensors by tailoring nanoparticle functionalization that could be applied to multiple electrolytes.

The implementation of this technology in the management of chronic kidney disease (CKD) has the potential to revolutionize treatment. Electrolyte disorders are common in CKD patients, given the dysregulation of kidney functions [19]. In the case of calcium, abnormalities in serum calcium can potentially lead to mineral and bone disorders [20], and cardiac arrhythmias [21]. However, currently available devices cannot be used for the continuous monitoring of electrolytes in CKD patients.

As mentioned, both the use of microporous materials, and the incorporation of nanoparticles inside the plasticized polymeric sensing films represent two emerging fields in the development of ion-selective electrodes, and their integration can bring potential advantages in terms of selectivity, and stability of devices. Within the present work, we report, for the first time, screening of different chemical functionalization of silica nanoparticles incorporated inside plasticized PVC-based ion sensors. This study allowed the determination of the impact of the interface between functionalized nanomaterials and polymeric films on ion sensing performance. Silica nanoparticles were chosen given their chemical versatility and the wide availability of coupling agents for their modification. Changes in mass absorption of calcium ions in solution and the electrochemical sensitivity of the films were measured and correlated to the water contact angle of functional groups present on the silica nanoparticles, leading to the rational design of a pre-concentration layer containing perfluoroalkane-functionalized silica nanoparticles. This layer could then be used to improve the selectivity of calcium ion sensors by tailoring the interaction of the films with the electrolytes, and minimized the loss of sensitivity over time by avoiding the leaching of sensing components.

Aerosol-Assisted Chemical Deposition (AACD) was used within the present work for the deposition of 5 µm thin nanocomposite sensing films containing the plasticized PVC matrix and the functionalized silica nanoparticles. This film showed selectivity towards Ca^2+^ ions and provided negatively charged sites to improve diffusion. The nanocomposite layer was deposited onto a ZrSiO_x_-based microporous material, allowing the fabrication of miniaturized sensing device. A ZrSiO_x_-based microporous nanomaterial was synthetized as sensing material by ionic imprinting. The combination of a silica-based nanocomposite deposited onto a microporous film allowed the selective diffusion and detection of Ca^2+^ ions. Device performance was tested in terms of the limit of detection and selectivity compared to other common electrolytes including Mg^2+^. The final device was also tested in a clinical environment, for the study of multiple ion dynamics during hemodialysis.

## 2. Materials and Methods

### 2.1. Materials

All reagents were purchased from Sigma Aldrich (St. Louis, MO, USA) unless otherwise indicated: Tetraethyl orthosilicate (98% purity), sodium tetraphenyl borate (≥99.5% purity), sodium ionophore X (94% purity), valinomycin (98% purity), magnesium ionophore I, zirconium acetylacetonate (97% purity), (3-glycidyloxypropyl) trimethoxysilane (≥98% purity), 1H, 1H, 2H, 2H-perfluorooctyl triethoxysilane (98% purity), 3[(trimethoxysilyl) propyl] diethylenetriamine (>85% purity), aminopropyl triethoxysilane (99% purity), trimethoxy(vinyl) silane (98% purity), (dimethylamino) trimethylsilane (97% purity), N-(trimethylsilyl) dimethylamine (97% purity), n-octyl triethoxysilane (97% purity), n-propyl triethoxysilane (98% purity), N,N-dimethyl trimethylsilylamine (97% sigma Aldrich), cyclodextrin (≥98% purity), ethanol (≥95% purity), high molecular weight PVC (Mw ~8000), bis(2-ethyl hexyl) sebacate (≥97.0% purity), (3-Aminopropyl)triethoxysilane (99% purity), calcium acetate (≥99% purity), p-toluenesulfonyl chloride (≥99% purity), sodium hydroxide (98% purity), 7 nm fused silica nanoparticles. Screen-printed electrodes were purchased from Saida Technology (Yaizu-shi, Japan).

### 2.2. Functionalization of Silica Nanoparticles

The standard reaction for the functionalization of silica nanoparticles has been reported elsewhere [22]. Briefly, 100 mg of 7 nm, as-received, silica nanoparticles were dispersed in 10 mL of methanol, and 30 mM of the coupling agent was added to the solution. The coupling agents employed in this study were (3-glycidyloxypropyl) trimethoxysilane (glycidyl), 1H, 1H, 2H, 2H-perfluorooctyl triethoxysilane (perfluorinated), aminopropyl triethoxysilane (APTES), trimethoxy(vinyl) silane (vinyl), trimethylsilane, N-(trimethylsilyl) dimethylamine (dimethyl amine), n-octyl triethoxysilane (n-octyl), n-propyl triethoxysilane (propyl), trimethyl(phenyl)silane (phenol), 3-[2-(2-aminoethylamino)ethylamino]propyl-trimethoxysilane (AAAPTS). The reaction was then incubated overnight at room temperature, and the nanoparticles obtained were washed 3 times in ethanol and cyclohexanone. 

### 2.3. Synthesis of Cyclodextrin-Functionalized Nanoparticles

Cyclodextrin-functionalized nanoparticles were also evaluated for the determination of the effects of large functional groups on the performance of the sensors. A protocol from Hsieh et al. was adapted for their synthesis [23]. A mixture of cyclodextrin and p-toluenesulfonyl chloride in a ratio of 2:1 mol in 220 mL water was mixed for 2 h. A solution of 40 mL NaOH 2.5 M was then added to trigger the reaction and the remaining p-toluenesulfonyl precipitates were removed. Ammonium chloride (10 g) was added to the solution, and the mixture was cooled to 4 °C and filtered. APTES (1.5 mmol) was then added to the suspension of mono-6-tosyl-β-CD, and the product then mixed with the 7 nm nanoparticles for their functionalization. The success of such functionalization was determined by FTIR (L160000A Perkin Elmer, Waltham, MA, USA).

### 2.4. Synthesis of Nanoporous Zirconium Silicate ZrSiO_x_ Nanoparticles

The ionically imprinted nanoparticles were synthesized in a pressure flask containing both the zirconium acetylacetonate and tetraethyl orthosilicate (TEOS) precursors at a 2:1 molar ratio. Firstly, 20 mM of tetraethyl orthosilicate (TEOS) were hydrolyzed by incubating them for 2 h in 10 mL of a 1:1 ethanol:water solution. Zirconium acetylacetonate in 10 mL of a similar solution containing 1:1 ethanol:water and a concentration of 40 mM was mixed with 10 mM of calcium acetate and added to the original TEOS solution. The calcium acetate provided the calcium ion templates for the creation of nanopores. The flask was then heated up to 250 °C and incubated for 8 h, and the temperature was subsequently lowered to 4 °C overnight. As a consequence of the low temperature, the components of the solution, including the acetylacetonate zirconium precursor, TEOS and calcium acetate changed to their minimum energy state, which led to the interaction of the calcium ions with the ionically imprinted zirconium silicate nanoparticles. However, due to the high presence of acetate groups in the zirconium precursor, a low reaction rate can potentially lead to the formation of gel-like products [24]. Thus, this process was repeated for at least 3 times to remove any acetate groups from the nanoparticles.

The obtained material was then characterized using SEM (EVO LS15, ZEISS, Jena, Germany), and EDS (Aztec, Oxford instruments, Oxford, UK) to characterize its morphology. In addition, FTIR was conducted to determine the presence of the Zr-O-Si bonds, powder XRD (L160000A Perkin Elmer, Waltham, MA, USA) was additionally used for the study of the crystalline structure of the nanoparticles, and the results were corroborated by HRTEM (FEG-TEM, JEM-2100F, JEOL, Tokyo, Japan) operating at an accelerating voltage of 120 kV. Finally, the pore size of the nanoparticles was measured by BET (Quantachrome, NOVATouch, Windsor, UK).

### 2.5. Device Fabrication

A membrane cocktail consisting of 31.5 mg PVC, 63.5 mg of bis 2-ethylhexyl sebacate (DOS) and 5 mg of the target nanoparticles were prepared using cyclohexanone as the carrying fluid. The functionalized silica nanoparticles had a double function in this study; firstly, they facilitated the diffusion of target ions inside the polymeric film, and then also acted as ionic sites, providing negatively charged surfaces. For the deposition of the films, 1 mL of the solution was employed, and deposited onto 50 nm thick gold electrodes using AACD through a pneumatic nebulizer. AACD was selected as the deposition method due to its ability to produce highly uniform, low-roughness films without the need for high temperatures or vacuum conditions. Compared to traditional casting methods, AACD offers improved film morphology, enhanced adhesion to the electrode surface, and better batch-to-batch reproducibility, while maintaining a low-cost and scalable fabrication process suitable for mass production. The incorporation of this method in the fabrication of ion-selective electrodes has been previously reported by our team, with successful results in the fabrication of PVC and nanocomposite-based electrochemical sensors [18]. 

**Figure 1:**
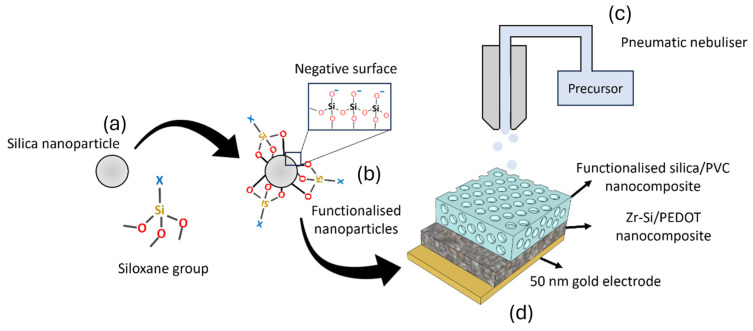
Schematic representation of the fabrication process of the sensing device. (**a**) Commercial silica nanoparticles were functionalized using different coupling agents. In all cases, siloxane groups were employed. (**b**) Functionalized silica nanoparticles were produced, with functional groups covalently attached to their surface. Silica nanoparticles also presented negatively charged sites that improved membrane diffusion processes. (**c**) A plasticized polymeric sensing film with silica nanoparticles was deposited by AACD using a pneumatic nebulizer. (**d**) Sensing film was deposited onto a sensitive layer containing Zr/Si nanoparticles, working as sensing agents. This whole device was deposited onto a 50 nm thin gold contact electrode that had been previously deposited onto a glass substrate.

The gold was plasma sputtered (Q150RES, Quorum technologies, Lewes, UK) onto a silica substrate. The AAVD deposition took place at 30–45 °C and the thickness of the obtained films could be evaluated using a stylus profilometer (Dektakxt, Bruker, Coventry, UK). In the case of ionically imprinted ZrSiO_x_ nanoparticles films, 1 mL of the solution obtained after the nanoparticle synthesis were centrifuged for 20 min at 4000 rpm and suspended in a solution containing 1.8% poly(3,4-ethylenedioxythiophene) (PEDOT). This mixture was then deposited onto a gold electrode at 80 °C. The process is illustrated in Figure 1.

### 2.6. Device Configuration

**Figure 2:**
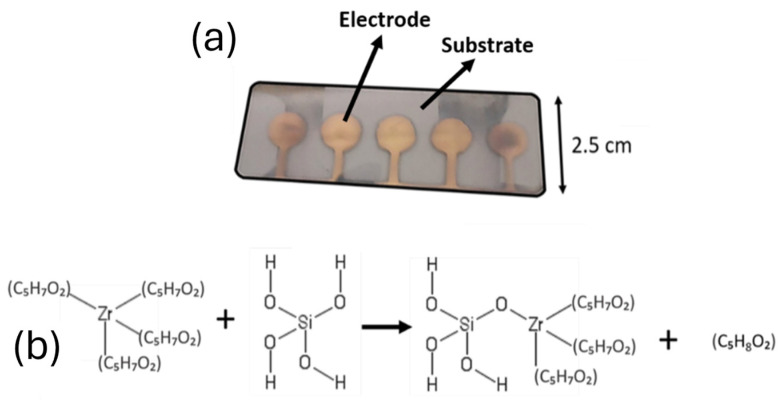
(**a**) Gold electrodes as deposited onto a glass slide. (**b**) Reaction between acetylacetonate and tetraethyl orthosilicate, as described in [26].

A novel device configuration was designed to improve the performance of all solid-state ion-selective electrodes used for the detection of calcium ions. This configuration incorporated the selectivity of perfluoroalkane-functionalized silica nanoparticles with ionically imprinted zirconium silicate nanoparticles. A sensing nanocomposite containing selective zirconium silicate porous nanoparticles and PEDOT polymer was deposited by AACD onto the gold electrode as the sensing elements (Figure 2a). PEDOT was chosen for the fabrication of the sensing nanocomposite layer due to its electrical conductivity and the ability to transduce the potentiometric signal derived from the adsorption of ions onto the selective zirconium silicate porous nanoparticles. A pre-concentration nanocomposite film containing 5 wt% of perfluoroalkane-functionalized silica nanoparticles was then deposited onto this conducting layer. This polymeric film was used as a pre-concentration membrane, facilitating the transport of Ca^2+^ and their interaction with the receptors. Zirconium-based compounds were selected in the form of zirconium acetylacetonate due to its capacity to form stable complexes with silicate and acetate precursors, facilitating the templating process through acetyl substitution chemistry (Figure 2b). Additionally, zirconium silicates were explored as novel adsorbents due to their structural stability and ability to interact with divalent ions [25].

### 2.7. Electrochemical Characterization of Films

For the assessment of the sensitivity of the synthesized electrodes, a three-electrode configuration was employed using a standard Ag/AgCl reference and platinum counter electrodes (Metrohm, Autolab BV, Oss, The Netherlands). Electrodes were pre-conditioned by immersing them in a 0.1 CaCl_2_ solution overnight. The open circuit potential (OCP) was then recorded at different concentrations of primary (Ca^2+^) or interference electrolytes (Na^+^, K^+^, Mg^2+^); 10^−8^, 10^−7^, 10^−6^, 10^−5^, 10^−4^, 10^−3^, 10^−2^, and 10^−1^ M. Sensor stability was tested both in terms of electrochemical noise and long-term stability. To characterize noise rates, electrodes were immersed in a 0.1 M concentrated CaCl_2_ solution for at least 1 h, and the standard deviation of electrochemical potential within a minute of operation was measured. Long-term stability was also determined as the increase of noise rates after 3 months of continuous contact of electrodes to a 0.1 M CaCl_2_ solution. As a control, a PVC membrane containing sodium tetraphenyl borate (NaTPB) instead of perfluoroalkane-functionalized nanoparticles was used.

Selectivity of the final devices was determined using a Fixed Interference Method (FIM), as described elsewhere [27]. Sensors were subjected to 0.1 M concentration of the interference ion, and the concentration of primary ion was increased. The intersection between the calibration plot containing both interference and primary ion and the one without primary ion was finally determined.

The lower detection limit of devices was calculated following IUPAC guidelines for ion-selective electrodes [28], as the lowest concentration of an analyte that can be reliably detected. This was calculated as the extrapolated activity of the analyte that meets the potential recorded on a blank solution as depicted in Figure S2. After calibration, this could be calculated analytically through the Nernst equation (Equation (1)):

(1) $$E=E_{0}-S\times log(C_{0})$$

where *E* is the base potential of the sensor at low concentrations, *E*_0_ is the reference potential, *S* is the slope of the sensors, and *C*_0_ is the detection limit. Limit of quantification, on the other hand, can be quantified as follow (Equation (2)):

(2) $$LOQ=10*(\frac{\sigma }{S})$$

where σ is the is the standard deviation of the response and *S* is the slope.

### 2.8. Quartz Microbalance Characterisation

The relative variations in the mass absorption of the films could be accurately evaluated using a quartz microbalance (Q-sense, Biolin Scientific, Västra Frölunda, Sweden). Different films containing 5 wt% of the abovementioned functionalized silica nanoparticles were deposited by AACD using the standard deposition conditions in terms of solvent composition and temperature as described in Section 2.4. In all cases, an initial incubation period was conducted on the nanocomposite thin films for 2 h in pure DI water (18 MΩ), followed by 2 h of incubation in 1 M of KCl or 1 M CaCl_2_. The relative ion intake was then calculated by taking the initial weight of the film after subjecting it to pure water and dividing it by the weight after using 1 M of the electrolyte solution (Equation (3)).

(3) $$RI=\frac{\left(W_{Ion}-W_{water}\right)}{W_{water}}$$

where *W_ion_* is the weight of the film after exposure to a solution containing 1 M of the target electrolyte (KCl or CaCl_2_) and *W_film_* represents the initial recorded weight of the film immersed in water.

### 2.9. Density Functional Theory Simulation of Materials

Interaction energies for the final material were studied by Density Functional Theory (DFT) using GAMESS software (https://chemcompute.org/gamess, accessed on 11 May 2025). A representative molecular structure was designed based on the stoichiometry observed from the EDS results, and 3D structure was optimized using Avogadro with a MMFF94 force field. A B3LYP/3-21G functional was employed. Similar models have been employed in the study of molecular interactions with porous materials, including zeolites [29]. Interaction energies were calculated as (Equation (4)):

(4) $$E_{interaction}=E_{ZrSiOx+Ion}-(E_{Ion}+E_{ZrSiOx})$$

where $E_{ZrOx+Ion}$ represents the energy of the system containing both ZrSiO_x_ and the target ion, $E_{Ion}$ is the energy of the ion, and $E_{ZrSiOx}$ is the energy of the ionically imprinted material, ZrSiO_x_.

### 2.10. Development of Electrolyte Sensors for Dialysate Sample Characterisation

Devices were tested using dialysate samples collected from stage 3 chronic kidney disease patients. For this testing, 4 different ion-selective electrodes were developed onto carbon-based screen-printed electrodes, with selectivity towards Na^+^, K^+^, Ca^2+^, and Mg^2+^. Carbon electrodes were used instead of the gold thin films given their low cost and wide commercial availability. Screen-printed electrodes were purchased and incorporated with a solid-state reference and counter electrodes. The use of these electrodes in the development of ion-selective electrodes have been widely reported, achieving a good performance.

Ca^2+^ selective electrodes were fabricated as described, using Zr/Si nanoparticles and perfluoroalkane-functionalized silica nanoparticles. Na^+^, K^+^, and Mg^2+^ were designed using a standard configuration, comprising a plasticized PVC film with DOS plasticizer. In all cases, 1 mg of ionophore (sodium ionophore X, valinomycin and magnesium ionophore I respectively) was added to a solution containing 33 mg PVC and 66 mg DOS. NaTPB was used in this case as an ionic site and added at 50 mol% of the ionophore. Electrodes were calibrated before being used, to ensure a Nernstian response.

### 2.11. Dialysate Collection and Analysis

A test trial of the sensing devices was conducted by immersing sensors in dialysate solutions collected from one patient with chronic kidney disease, as part of an observational pilot study approved by UK National Research Ethics committee, 21-NI-0059. Informed consent was obtained before the trial. Dialysate samples were collected from the waste output of a hemodialysis filtration system at 4 different times (5, 30, 60, and 120 min). Electrodes were immersed directly into dialysate solutions and potentiometric response was recorded continuously for at least 5 min on each case. 

## 3. Results and Discussion

### 3.1. Study of the Sensitivity and Selectivity of the Ion Sensors

**Figure 3:**
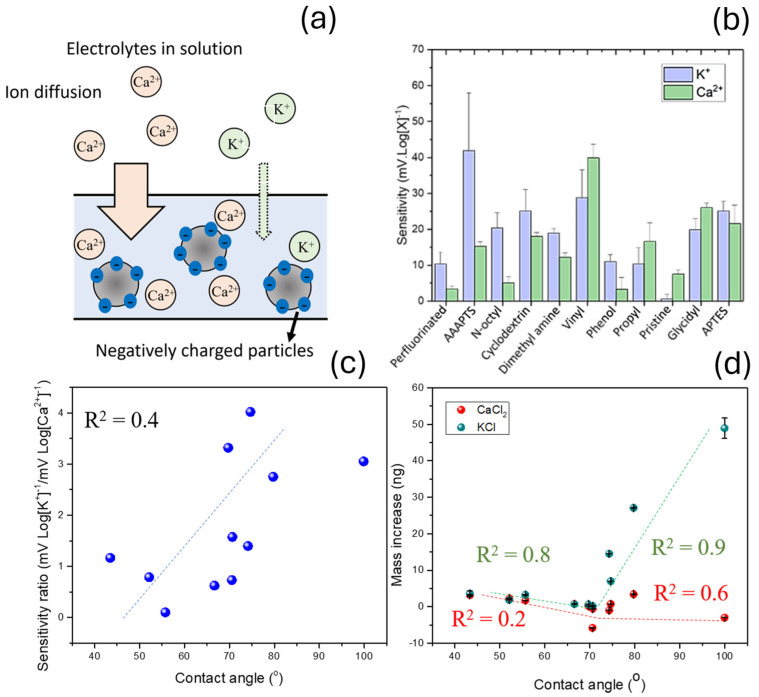
(**a**) Schematic representation of sensing concept. Ca^2+^ selectively diffuse into sensing membrane due to the presence of negatively charged functionalized nanoparticles. (**b**) Absolute values of sensitivity towards monovalent (K^+^) and divalent (Ca^2+^) ions upon doping of PVC membranes with different coupling agents, as indicated in the x-axis. (**c**) Sensitivity ratio between calcium and potassium responses as a function of contact angle found in functional groups. (**d**) Relative mass absorption of films in presence of KCl (green) and CaCl_2_ (red) against hydrophobicity of functional groups previously described. R^2^ values of plots are indicated.

Unspecific diffusion of electrolytes including potassium ions inside polymeric films is considered as one of the major limitations in ion-selective electrodes for calcium detection. This effect can decrease the selectivity of the sensors by triggering a potentiometric signal in the presence of the non-target analytes [30]. To test the impact of different silica functionalization on the perm-selectivity of plasticized PVC membranes, multiple devices were fabricated incorporating the same PVC/DOS composition (1:3), and 5 wt% of functionalized silica nanoparticles. These nanoparticles showed a double function, by providing ideal conditions for ion diffusion and adding negatively charged ionic sites to attract cations (Figure 3a). Initially, differences in sensitivity of the sensors towards K^+^ and Ca^2+^ separately without any sensing material embedded in the membranes were quantified. Ten different siloxane functional groups were tested in this study (Figure 3b), listed in Figure S1. The theoretical hydrophobicity of the functional groups was additionally calculated. Flat silica substrates were functionalized as conducted in our previous work [18]. The silica substrate was subjected to the same functionalization process of the silica nanoparticles and their water contact angle was measured. As such, this measurement could be used to estimate the water contact angle of the surface of these functional groups. Water contact angle obtained after using functionalized flat silica surfaces could be correlated with the sensitivity obtained in the case of potassium and calcium ions (Figure 3c). The ratio between the sensitivity towards calcium and potassium was calculated, to reflect the trends in increased Ca^2+^ diffusion over K^+^. Moreover, the changes in mass of the films were determined, which provided further evidence of the enhanced perm-selectivity properties of films (Figure 3d).

In general terms, the ratio between the sensitivity achieved for K^+^ and Ca^2+^ increased with the hydrophobicity of functional groups, with a relatively high correlation coefficient (R^2^ = 0.4). A low sensitivity ratio between the sensitivity obtained in the case of potassium ions and calcium ions was observed when pristine silica nanoparticles were incorporated in the sensing nanocomposites. This material showed a water contact angle of 55° as measured by the flat silica surface model, and the sensitivity ratio was 0.08 mV Log[K^+^]^−1^/mV Log[Ca^2+^]^−1^. In the case of 3-[2-(2-Aminoethylamino)ethylamino]propyl-trimethoxysilane (AAAPTS), cyclodextrin, and vinyl, a near-Nernstian response towards both ions was measured in absolute values. This fact was attributed to the direct interaction of these groups with both the K^+^ and Na^+^, since both compounds can be used as ionophores for the detection of cations [31,32]. However, the ratio between the sensitivity towards K^+^ and Ca^2+^ in these sensors remained within the observed linear trend. The sensitivity towards K^+^ and Ca^2+^ ions on pristine plasticized PVC membranes without the use of nanoparticles or ionic sites was additionally measured as a control, with 9 ± 1 mV Log[K^+^]^−1^ and −4 ± 4 mV Log[Ca^2+^]^−1^, respectively. The low sensitivity achieved in this case, reaching −4 ± 4 mV Log[Ca^2+^]^−1^, was a consequence of the lack of negatively charged sites inside the membrane, provided by the silica nanoparticles.

The ionic absorptive behaviour of the nanocomposites was further characterized by a quartz microbalance. This tool allowed the determination of the total ionic concentrations in terms of mass absorption instead of the activity of the K^+^ and Ca^2+^ present inside the polymeric membrane as calculated by the potentiometric signals. Consequently, the diffusion of both positively and negatively charged ions diffusing inside the nanocomposite sensing films could be quantified. This effect of simultaneous diffusion of positively and negatively charged ions in the sensing films is often disregarded in the literature, and it is normally neglected in most simulation studies [33]. However, counterions play a pivotal role in the maintenance of a good performance in the ion-selective electrodes, being essential for the transduction mechanism of some conducting polymers [34] and can decrease the sensitivity of the electrodes due to their opposite charges.

In this study, quartz microbalance was used to determine the changes in mass of the sensing films deposited onto the electrodes. Such changes in mass showed a behaviour similar to the previous case of electrochemical selectivity. A higher absorption of both Ca^2+^ and K^+^ was observed when functional groups with a high contact angle such as perfluoroalkane or AAAPTS where employed. In the case of Ca^2+^, the weight increase reached a maximum at 99 ± 1°, with 49 ± 3 ng_ion_ and was minimal at 70 ± 3°, being 0.13 ± 0.04 ng_ion_, while in the maximum in potassium absorption was found at 79.7 ± 0.7° with 3.41 ± 0.01 ng_ion_ and the minimum −5.81 ± 0.01 ng_ion_ at 70.6 ± 0.4°. Thus, a preferential intake of CaCl_2_ over KCl was observed in the case of sensing nanocomposites containing perfluoroalkane-functionalized silica nanoparticles. However, even though the absorption of Ca^2+^ ions was significantly higher in the case of perfluoroalkane functionalization, the absolute electrochemical sensitivity of the sensing nanocomposite previously measured was one of the lowest results among all the functionalized sensors. Thus, this improvement on the ion diffusivity could not be explained by the uptake of charged cations alone, since the high absorption of calcium ions would have been translated into a near-Nernstian signal. As such, this effect was attributed to a higher anion permeability parallelly to the Ca^2+^ uptake. The enhanced anion permeability would increase the total amount of absorbed ions, since both Cl^−^ and Ca^2+^ diffuse into the nanocomposite sensing films, but it minimized the total potentiometric signal since the charge from calcium ions is countered by Cl^−^ ions. This selective membrane, which incorporates the perfluoroalkane silica nanoparticles, could serve as a pre-concentration layer to enhance the selectivity of sensing materials, by allowing the diffusion of Ca^2+^ specifically.

### 3.2. ZrSiO_x_ Particle Characterization

**Figure 4:**
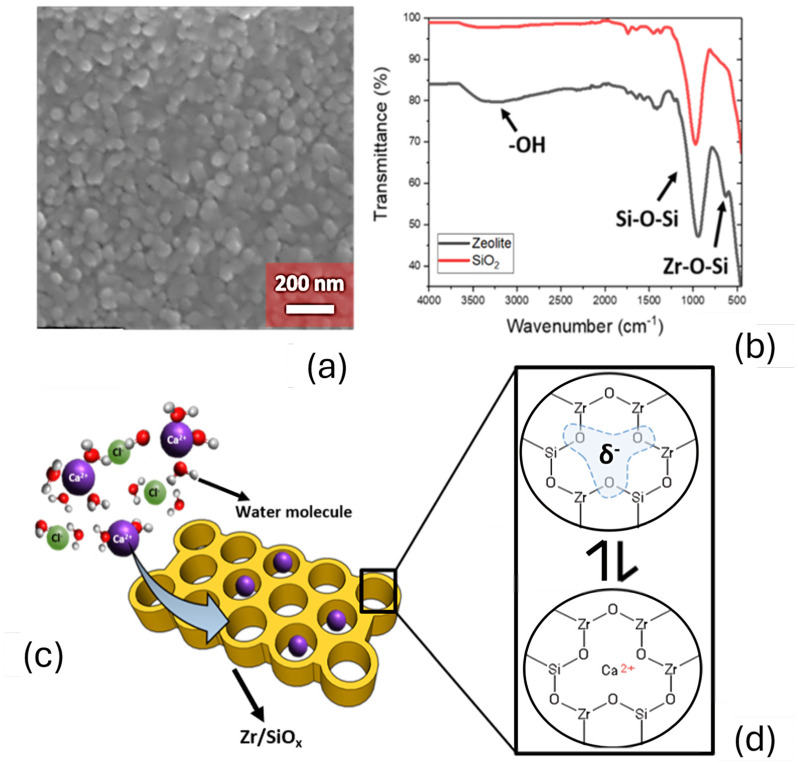
(**a**) SEM imaging of zirconium silicate nanoparticles. (**b**) FTIR analysis of silica (red) and ionically imprinted ZrSiO_x_ nanoparticles (black), showing characteristic peaks of Si-O-Si and Zr-O-Si present in the sample. (**c**) Schematic representation of operation principle of ionically imprinted zirconium silicate nanoparticulate material, showing a porous structure that allows adsorption of electrolytes. (**d**) The characteristic pore shape and chemical properties allow the specific interaction with Ca^2+^ due to the negative environment around the pores, driven by the oxygen bridges and the pore size.

Ionically imprinted zirconium silicate nanoparticles presented a spherical morphology with a size in the range of circa 50 nm (Figure 4a). The composition of nanoparticles was confirmed by EDS, revealing the presence (in atomic percentages) of 9 at% of Zr, 17 at% of Si, and 64 at% of O, being consistent with the formulation used for the ionically imprinted ZrSiO_x_ nanoparticles. To confirm the chemical structure of the ionically imprinted ZrSiO_x_ nanoparticles, an analysis by FTIR was conducted (Figure 4b). FTIR revealed the presence of a 1100 cm^−1^ stretch due to the presence of Si-O-Si bonds, and an additional stretch at 700 cm^−1^, corresponding to Zr-O-Si as reported [35,36,37]. This particular structure and composition allowed an ionic adsorption by the direct interaction with the molecular pores present in the nanoparticles (Figure 4c,d).

**Figure 5:**
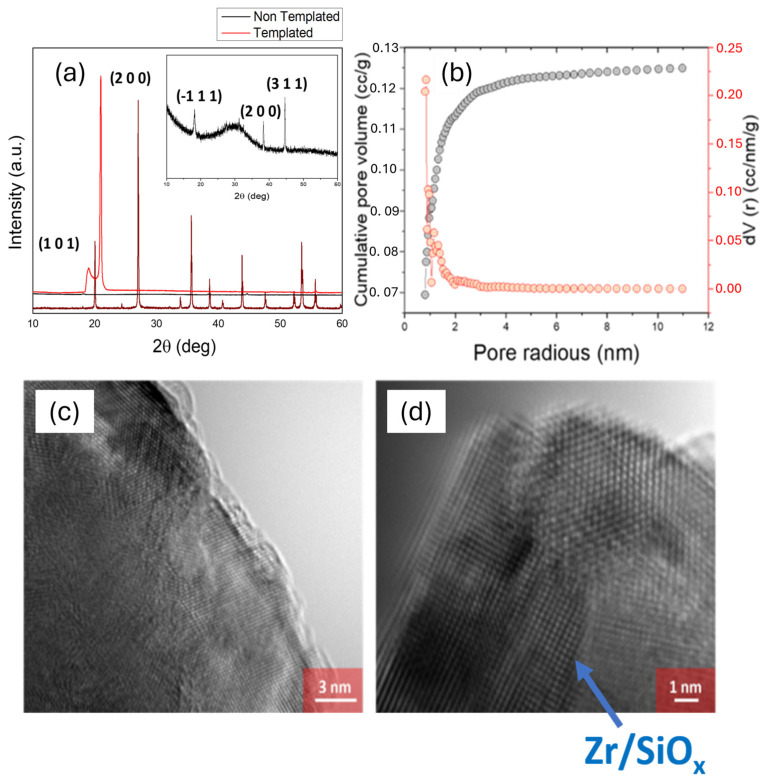
(**a**) XRD pattern of ionically imprinted (red) and non-templated (black) ZrSiO_x_ nanoparticles, showing 2θ peaks at 19° and 21° (JCPDS card no. 00-006-0266). (**b**) BET results showing cumulative pore, volume, and differential plot of ionically imprinted ZrSiO_x_, with a higher presence of pores in the range of 0.84 nm. (**c**,**d**) HRTEM visualization of the structure found in the ionically imprinted zirconium silicate nanoparticles at two different magnifications.

The next step on the characterization involved the study of the morphology and crystal structure of ionically imprinted nanoparticles. Powder XRD was used to study the crystal structure of the nanoparticles. Interestingly, when calcium was present during synthesis, the XRD pattern of the resulting material displayed major diffraction peaks at 19.22° and 20.97°, along with minor peaks at 56.39° and 62.07° (Figure 5a). These features resembled those reported for calcium zirconium silicates, suggesting that Ca^2+^ ions promote the crystallization of a distinct calcium zirconosilicate structure. It is worth noting that while zircon (ZrSiO_4_) typically exhibits its strongest diffraction peaks at higher angles (2θ circa 26.6° and 30.2°), the emergence of strong low-angle peaks (19–21°) indicate the formation of an alternative phase, potentially associated with amorphous or low-crystallinity zirconium silicate-type materials. This behaviour aligns with the ionic imprinting methodology, where the introduction of specific ions during synthesis would direct the assembly of the material at the nanoscale.

This pattern did not correspond with the XRD spectrum of the ZrO_2_ or SiO_2_. However, the observed peak at 2θ of 21° was consistent with the (2 0 0) plane typically found in zircon (ZrSiO_4_), at 27° [38], with a shift due to the presence of Si atom substitutions. The pore radius of this zirconium silicate material was additionally studied by BET, being in the range of 0.84 nm (Figure 5b). 

To study the effect of templating nanomaterials during synthesis using Ca^2+^ ions, ZrSiO_x_ nanoparticles were prepared without the use of CaCl_2_. In this case, the XRD pattern showed a combination of the cubic phase of zirconium oxide [39] and amorphous silica. The morphology of the final material after exposure to calcium ions could be observed at an atomic scale using HRTEM (Figure 5c,d). The HRTEM revealed an ordered arrangement of the atoms, being consistent with the crystalline structure of ZrSiO_x_ as reported by XRD. Interplanar spacing was calculated from the HRTEM results using ImageJ, Version 1.54p (Figure S3). The calculated spacing was 0.51 nm, which was aligned with the one calculated using Bragg’s law on the XRD diffraction peaks (0.47 and 0.41 nm). On the contrary, the interplanar spacing for (1 0 0) and (2 0 0) planes in zirconium silicate are 0.33 and 0.44 nm. These observations suggest the successful incorporation of Ca^2+^ during synthesis, leading to a slight expansion and rearrangement of the zirconium silicate lattice. This nanoscale structural modification supports the enhanced selectivity towards Ca^2+^ as observed in electrochemical measurements, demonstrating that ionic imprinting has affected both the crystallinity and sensing performance of the material.

**Figure 6:**
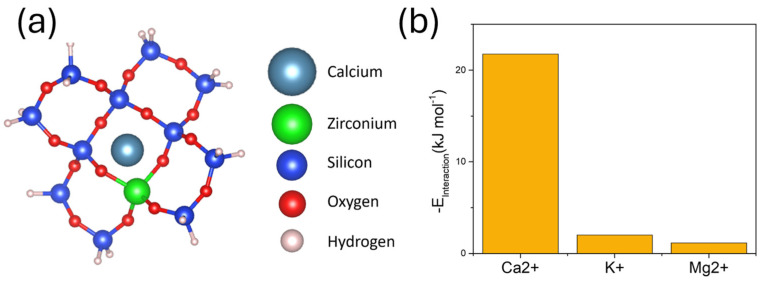
(**a**) 3D structure of ZrSiO_x_ structure interacting with Ca^2+^ ions. (**b**) Calculated interaction energy of ZrSiO_x_ with different cations, including Ca^2+^ (target ion), and main interferences (Mg^2+^, K^+^).

The porosity of ionic receptors is crucial to ensure a proper selectivity of the sensors. This parameter has been observed to change sensing capabilities of multiple aluminosilicates, where a change in the pore size can be obtained by changing the ratio between aluminium and silicon elements. This concept was demonstrated by Chudasama et al. [40] who increased the adsorption of nitrogen gas on aluminosilicate zeolites by increasing the content of silica on this nanomaterial. The increase in the content of silica could successfully increase the pore size of the zeolites, enhancing the absorption capabilities. In the case of ion sensors for the detection of calcium ions, a high porosity combined with the chemical composition of the ionically imprinted zirconium silicate nanoparticles are desired for enhancing the adsorption of Ca^2+^ ions. Moreover, the magnitude of the pore size achieved in this study (0.84 nm) is similar to other reported zirconium cyclosilicates, currently used for the extraction of specific ions such as K^+^ [41]. This size was also slightly higher than the one reported for calcium ions (0.11 nm), allowing a more effective interaction between pore elements and the ions (Figure 6a). The strength of this interaction was stronger when compared to similar ions, such as magnesium ions, with a smaller ionic radius of 0.08 nm, leading to a high selectivity. Differences in interaction energy could be studied using DFT. Lowest energy was calculated in the case of Ca^2+^ interaction with ZrSiO_x_ as expected, with E_interaction_ = −21.77 kJ mol^−1^. This negative energy was over an order of magnitude lower than the energy observed in the case of main interferences K^+^, and Mg^2+^ that showed interaction energies of −2.01 and −1.16 kJ mol^−1^ respectively. Interaction energy with Na^+^ was additionally calculated. However, in this case, interaction energy was +73.25 kJ mol^−1^, which led to a low sensitivity.

### 3.3. Electrochemical Characterization of the Ion Sensing Devices

**Figure 7:**
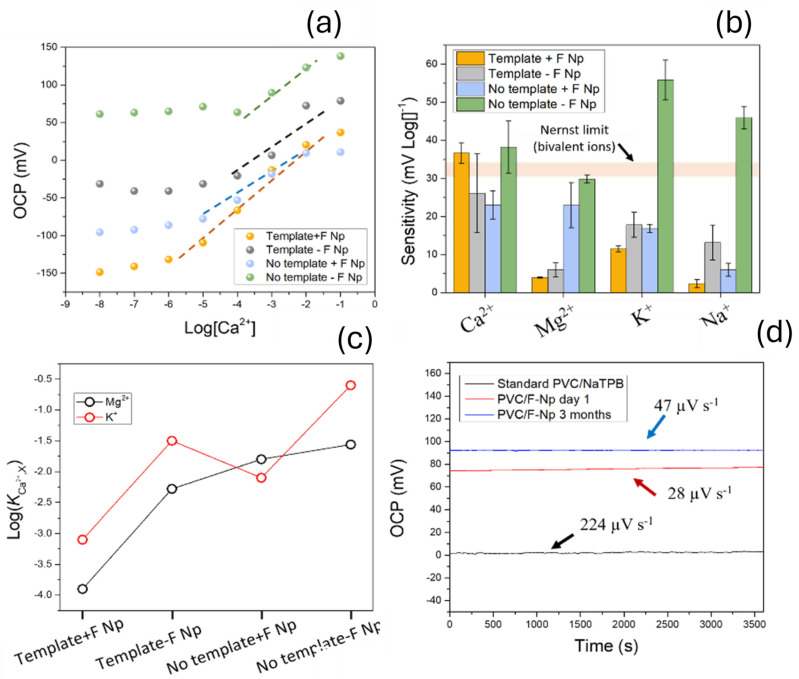
(**a**) Calibration plot of developed electrodes using different concentrations of Ca^2+^ and Mg^2+^ ions. Results for ionically imprinted ZrSiO_x_ and F-functionalized silica nanocomposite (Template + F-Np), ionically imprinted ZrSiO_x_ and no F-functionalized silica nanocomposite (Template–F-Np), not templated ZrSiO_x_ and F-functionalized silica nanocomposite (No template + F-Np), and not templated ZrSiO_x_ and no F-functionalized silica nanocomposite (No template–F-Np), are shown. F-functionalized silica nanoparticles denote perfluoroalkane-functionalized silica nanoparticle embedded in plasticized PVC film. (**b**) Comparison of sensitivities obtained using full sensor set up, containing ionically imprinted zirconium silicate nanoparticles and pre-concentration layer containing perfluoroalkane-functionalized silica nanoparticles (orange), ionically imprinted zirconium silicate nanoparticles with no perfluoroalkane-functionalized silica nanoparticles incorporated into the PVC membrane (green) and full device with pre-concentration layer with perfluoroalkane-functionalized silica nanoparticles and non-templated zirconium silicate nanoparticles (blue). Highlighted area in orange indicates standard Nernst sensitivity for monovalent ions. (**c**) Selectivity coefficients for K^+^ and Mg^2+^ using Fixed Interference Method. (**d**) Potentiometric signal recorded continuously for 1 h to calculate noise rates before and after 3 months of continuous exposure to 0.1 M CaCl_2_.

The performance of the developed sensors was characterized by recording the open circuit potential of the electrodes over time at various concentrations of calcium, magnesium, potassium and sodium ions. A 5 µm thick film of the plasticized PVC with the perfluoroalkane-functionalized nanoparticles was deposited onto the sensing film, consisting of ZrSiO_x_ embedded inside a PEDOT matrix, by AACD. The role of each component (plasticized PVC film, templating during ion imprinting process) was tested by fabricating sensors and determining their performance (Figure 7a). The sensitivity of the sensors towards calcium, magnesium, potassium, and sodium ions was determined. Each electrolyte was tested using separate solutions with different concentrations; 10^−8^, 10^−7^, 10^−6^, 10^−5^, 10^−4^, 10^−3^, 10^−2^, and 10^−1^ M. Devices were tested against Ca^2+^, Mg^2+^, K^+^, and Na^+^, which are the most common interferences for ion-selective electrodes, and they are ubiquitously present in all body fluids.

The sensitivity towards Ca^2+^ of the full devices was super-Nernstian, in the range of 37 ± 3 mV Log[Ca^2+^]^−1^, which was higher compared with the Na^+^ (2 ± 1 mV Log[Na^+^]^−1^), K^+^ (11 ± 1 mV Log[K^+^]^−1^), and Mg^2+^ (4 ± 1 mV Log[Mg^2+^]^−1^) which all showed a sub-Nernstian behaviour (Figure 7b). The super-Nernstian sensitivity observed was a consequence of the electrostatic interactions between Ca^2+^ ions and the ZrSiO_x_ surface, and high surface area of ZrSiO_x_ nanomaterials. This super-Nernstian behaviour has been observed in silicon-based nanomaterials used as cation sensors [42], and potentiometric sensors based on ionically imprinted silica nanoparticles [43].

The sensitivity profile towards the main (Ca^2+^) and interference ions (K^+^, Mg^2+^) was reflected on low selectivity coefficients in the full device, containing ZrSiO_x_, and F-functionalized silica nanoparticles $k_{{Ca}^{2+},\;K^{+}}^{FIM}=-3.1$ and $k_{{Ca}^{2+},\;{Mg}^{2+}}^{FIM}=-3.9$. These coefficients increased upon removing different elements in the device, including the templating, and PVC films (Figure 7c). These values are lower than other currently reported coefficients for ionophore-based ion-selective electrodes, circa $k_{{Ca}^{2+},\;{Mg}^{2+}}^{FIM}=-3.1$ and $k_{{Ca}^{2+},\;K^{+}}^{FIM}=-2.6$ [44,45]. On the contrary, when no perfluoroalkane-functionalized Si nanoparticles were incorporated, the sensitivity towards K^+^ increased up to 18 ± 3 mV Log[K^+^]^−1^, and the selectivity coefficients increased to −2.3 and −1.5 for Mg^2+^ and K^+^ respectively. In this case, ionic sites were incorporated via 0.5 wt% NaTPB to promote the diffusion of anions. These results demonstrated the impact of including a pre-conditioning layer on the surface of nanoparticles. Moreover, the effects of templating nanoparticles were determined by fabricating a sensor incorporating perfluoroalkane-functionalized Si nanoparticles but without including Ca^2+^ during the synthesis of ZrSiO_x_ sensing nanoparticles. In this case, the sensitivity of devices towards Mg^2+^ increased to a similar value of Ca^2+^, with 23 ± 6 mV Log[Mg^2+^]^−1^. When no templating or perfluoroalkane-functionalized Si nanoparticles were incorporated in the film, a near-Nernstian sensitivity was achieved in all cases.

Prior to the testing of the sensitivity of the ion sensors containing the ionically imprinted zirconium silicate nanoparticles, nanocomposites were pre-conditioned overnight in a solution containing 0.1 M of CaCl_2_. The response signal during this initial conditioning of the electrodes was then recorded and used to study the drift stability of the sensors. Sensors showed a low electrochemical noise of 97 µV s^−1^ being lower than the noise achieved by sensors with a standard composition, without including nanoparticles, in the range of 115.6 µV h^−1^ [18], and reaching up to 900–200 µV h^−1^ [46,47]. When only the ionically imprinted zirconium silicate nanoparticles in the PEDOT polymeric matrix were deposited onto the contact electrode and no pre-concentration nanocomposite film was used, a high noise in the range of 2.79 mV h^−1^ was observed, and with a low sensitivity of 10 ± 2 mV Log[Ca^2+^]^−1^.

The long-term performance of devices was finally characterized by immersing sensors in 0.1 CaCl_2_ solutions for 3 months. Initial electrochemical noise in this case was significantly lower than the one achieved by using standard membranes containing NaTPB-based films, with 28 µV s^−1^ and 224 µV s^−1^, respectively. After 3 months of continuous operation, the noise rate increased to 47 µV s^−1^, lower than the standard NaTPB films (Figure 7d). One of the main drivers of performance loss in ion-selective electrodes is the migration of active components, including ionophores in the field to the sample solution over time [12]. The present device structure incorporates both ionic sites and selective components with solid nanoparticles, minimizing any leaching. However, a leaching of plasticizers has also been observed in PVC-based ion-selective electrodes [27]. The use of plasticizers is key to enable ions to flow to the sensing film [48]. As such, leaching can impact the performance of devices in the long-term. The incorporation of functionalized silica nanoparticles can mitigate this effect, even allowing endurance under high pressure conditions [18]. The long sensor lifetimes achieved in this case could then be explained given the interactions between matrix components and functionalized silica nanoparticles.

The results from electrochemical characterization demonstrated the improved performance of ZrSiO_x_ nanoparticles when used in combination with perfluoroalkane-functionalized nanoparticles. These devices showed an improved selectivity and electrochemical noise rates. Sensors also showed a low limit of detection, within the micromolar range (~1 µM) and sub-micromolar level limit of quantification (7 µM).

### 3.4. Clinical Testing in Dialysate Samples

Devices were finally tested in a clinical environment to evaluate the feasibility of using sensors for monitoring patients with chronic kidney disease. Devices were fabricated using screen-printed electrodes to lower production costs. In all cases, a Nernstian sensitivity of sensors was achieved. In the case of Ca^2+^ devices, sensitivity achieved was similar, being 35 mV Log^−1^ and with noise levels increased slightly to 48 µV s^−1^.

**Figure 8:**
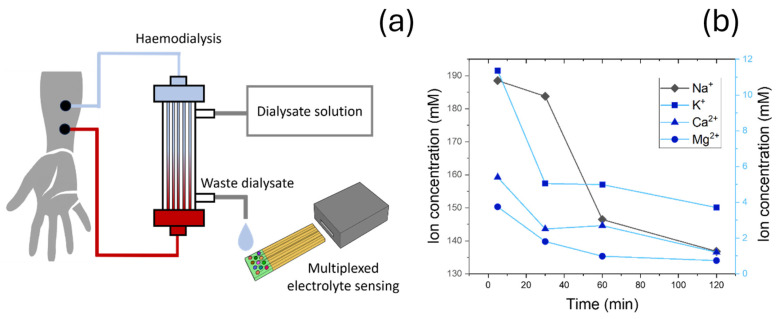
(**a**) Schematic representation of dialysis process. (**b**) Waster dialysate samples were taken at 5, 30, 60, and 120 min, and electrolyte profile was determined by ion-selective electrodes.

Dialysate effluent samples from end-stage chronic kidney disease patients during routine haemodialysis treatments were collected from the outflow of a haemodialysis equipment (Figure 8a) at four different times (5, 30, 60, 120 min), and ion-selective electrodes were directly immersed into each sample. Dialysate is the fluid used in haemodialysis to remove waste products and excess substances from the blood. Typical dialysate membranes only allow relatively small molecules (with a maximum size around 500 Da [49]), while large molecules such as proteins are minimally filtered into the dialysate. The use of functionalized silica nanoparticles can further mitigate fouling effects. Sensors containing silica nanoparticles have been shown to reduce protein fouling, achieving good performance even in human serum [18]. As such, potential interference with sensor measurements due to fouling are not expected.

For this study, four different electrodes were fabricated, with selectivity towards the main serum electrolytes (Na^+^, Ca^2+^, K^+^, Mg^2+^). In the case of Ca^2+^, devices were fabricated as described, using ZrSiO_x_ nanoparticles and a perfluoroalkane-functionalized silica nanoparticle-based top layer. Na^+^, Mg^2+^, and K^+^ sensors were developed using a standard composition, with a plasticized PVC film, ionophores, and using 50 mol% of sodium tetraphenyl borate as ionic site. Ionophores used in this study were sodium ionophore X, magnesium ionophore I, and valinomycin.

The characterization of clinical samples demonstrated a decrease in all four electrolytes over time, which reached a plateau around 120 min after the dialysis procedure started. In all cases, electrolytes stabilized around the normal concentrations found in serum. As expected, sodium represents the main electrolyte, with an initial concentration around 188.25 mM (clinical concentration 135–142 mEq L^−1^ [50]). This high concentration was attributed to the high physiological concentrations, and minor effects from interference electrolytes present initially in the sample in high concentrations (i.e., K^+^, positively charged toxins). In the case of K^+^, Mg^2+^, and Ca^2+^, changes in concentrations were significantly lower, given their small quantities in serum. Clinical concentration ranges are 3.5–5.5 mEq L^−1^ for K^+^ [51], 0.75–0.95 mmol L^−1^ in the case of Mg^2+^ [52], and 1.1–1.3 mmol L^−1^ for free Ca^2+^ [53], which corresponds to a total concentration in the range of 2.2–2.7 mmol L^−1^ [54]. While clearance dynamics for each electrolyte varied, final concentration values measured for Na^+^, K^+^, and Mg^2+^ were within the standard clinical values, with 136.98, 3.72, and 0.83 mM respectively. Ca^2+^ concentrations were 1.19 mM, which was also in the range of standard physiological concentration for Ca^2+^. Maintaining these concentrations is key since low concentration of Ca^2+^ in serum has been reported to lead to cardiovascular complications and sudden cardiac arrest, given the involvement of this ion in cardiac conduction and muscle function [55].

As such, the final sensors could be used in combination with selective electrodes with selectivity towards other key electrolytes (Na^+^, K^+^, and Mg^2+^) for the profiling of ions in chronic kidney disease patients undergoing haemodialysis treatments. Samples at different times (5, 30, 60, and 120 min) were collected, and devices were used to quantify the changes in electrolytes over time. In all cases, electrolytes reverted to physiological levels after 120 min.

## 4. Conclusions

Within the present work, a new Ca^2+^-selective sensor has been designed using a two-layer composite film approach. This novel configuration, incorporating a ZrSiO_x_ nanomaterial and a perfluoroalkane-functionalized silica nanoparticle-based layer, led to a Ca^2+^ sensor with high selectivity and electrochemical stability. 

A systematic study of the effects of different silica functionalizations on the performance of ion-selective electrodes was conducted for the first time. This study incorporated a quartz microbalance characterization, which provided further insights into ion dynamics, and revealed a favoured diffusion of Ca^2+^ and Cl^−^ counterions which could not be observed through standard OCP potentiometry. Functionalized silica nanoparticles had an impact on the final membrane structure and provided ionic sites for ion sensing.

A ZrSiO_x_ nanomaterial was additionally synthesized and characterized. Its crystalline structure and chemical composition were confirmed by XRD and EDS, respectively. Moreover, BET revealed a high porosity, with a mean pore size in the range of 0.84 nm. This material could selectively bind Ca^2+^ over Mg^2+^, K^+^, and Na^+^. 

The final device had a thickness of 5 µm. The impact of incorporating both the pre-concentration layer and templating ZrSiO_x_ nanoparticles during synthesis was studied through the determination of sensitivity and selectivity coefficients. When full devices were developed, a super-Nernstian sensitivity of 37 ± 3 mV Log[Ca^2+^]^−1^, a low response time (>10 s) and a good selectivity in the range of $k_{{Ca}^{2+},\;K^{+}}^{FIM}=-3.1$ and $k_{{Ca}^{2+},\;{Mg}^{2+}}^{FIM}=-3.9$ were achieved. Devices could be used continuously for at least 3 months while maintaining an ultra-low noise rate lower than 47 µV s^−1^, one of the lowest reported for ion-selective electrodes. This stability was attributed to the use of ZrSiO_x_ nanoparticles instead of small molecules as ionophores, reducing the leaching to sample solution over time. 

These results reflect the need for developing new sensor systems that can simultaneously quantify multiple electrolytes in patients with chronic kidney disease. These systems would have the potential to improve patient health by optimising dialysate compositions, and providing tailored recommendations for treatment options.

## Data Availability

Data is contained within the article or Supplementary Materials.

## Supplementary Materials

The following supporting information can be downloaded at: https://www.mdpi.com/article/10.3390/nano15100741/s1, Figure S1. Table containing the molecular structures of the coupling agents employed for the functionalisation of the silica nanoparticles and their respective contact angles. Figure S2. Schematic depiction of process to quantify detection limit. Figure S3. Calculation of interplanar spacing from HRTEM images.
