# Supplementary material for: Calcium Ion Sensors with Unrivaled Stability and Selectivity Using a Bilayer Approach with Ionically Imprinted Nanocomposites
Source: Nanomaterials (Basel). 2025 May 15;15(10):741. doi: 10.3390/nano15100741 (PMC12114124; doi:10.3390/nano15100741)
Supplement: Supplementary file 1 [file nanomaterials-15-00741-s001.zip › nanomaterials-3573895-supplementary.pdf]

Supplementary materials

Name	Contact angle	Group
Perfluoroalkane	100.0±1.3	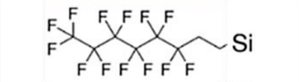
AAAPTS	79.7±0.8	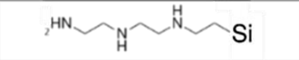
n-Octyl	74.6±0.8	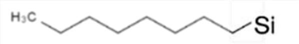
Cyclodextrin	74.3±2.9	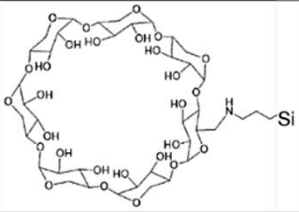
Dimethyl amine	70.6±2,8	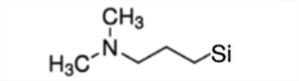
Vinyl	10.4±0.6	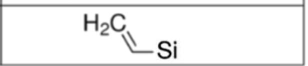
Phenol	69.7±0.7	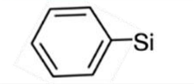
n-Propyl	66.6±0.5	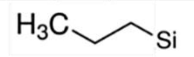
Pristine (Control)	55.7±0.3	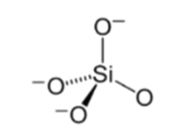
Glycidyl	52.1±1.2	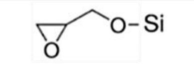
APTES	43.3±1.4	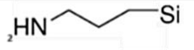

Figure S1. Table containing the molecular structures of the coupling agents employed for the functionalisation of the silica nanoparticles and their respective contact angles.

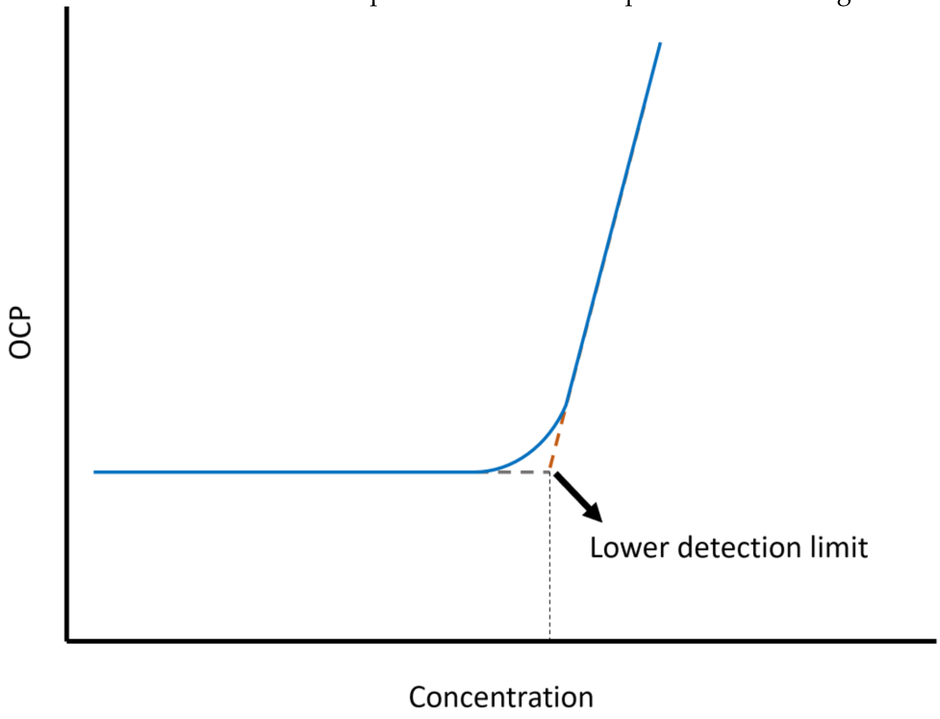

Figure S2. Schematic depiction of process to quantify detection limit.

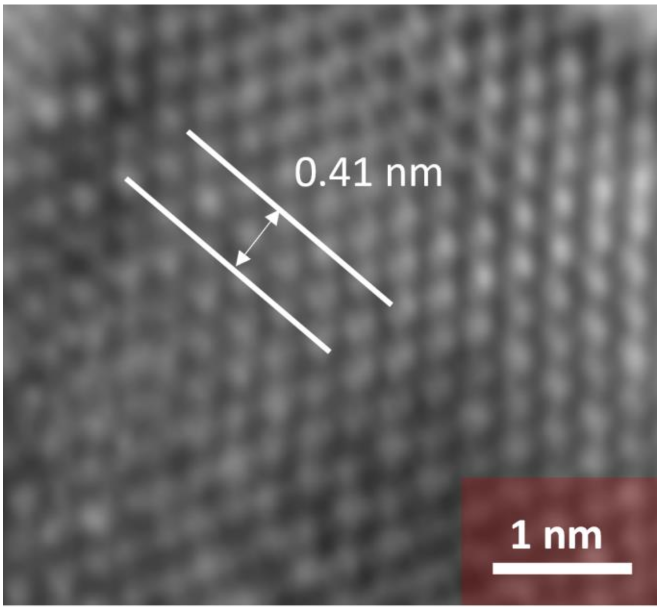

Figure S3. Calculation of interplanar spacing from HRTEM images.
